# STANDARDIZED NURSING LANGUAGE—KNOWLEDGE-BASED CLINICAL DECISION SUPPORT SYSTEM MODULE FOR LONG TERM CARE NURSES

**DOI:** 10.1093/geroni/igae098.4081

**Published:** 2024-12-31

**Authors:** Juh Hyun shin, Chung Hyuk Park, Myungeun Lee, Soo-Kyoung Lee, Crystel Farina, Suhyun Park, Burton Korer, Melissa Batchelor

**Affiliations:** George Washington University, Washington, District of Columbia, United States; George Washington University, Washington, District of Columbia, United States; George Washington University, Washington, District of Columbia, United States; Seoul National University, Seoul, Republic of Korea; George Washington University, Washington, District of Columbia, United States; University of Minnesota, Minneapolis, Minnesota, United States; George Washington University, Washington, District of Columbia, United States; George Washington University, Washington, District of Columbia, United States

## Abstract

Clinical decision support systems (CDSS) integrate patient health information with clinical expertise to guide referrals, reduce prescription medication errors, and ensure adherence to guidelines. Standardized nursing languages, such as NANDA-I (North American Nursing Diagnosis Association International), NIC (Nursing Interventions Classification), and NOC (Nursing Outcomes Classification), enhance evidence-based practice by providing essential diagnostic and outcome data. However, the adoption of CDSS in long term care settings, incorporating standardized nursing languages and culturally specific scenarios to improve care for diverse racial and ethnic groups. We employed machine learning techniques, including support vector machines, random forests, and large language modeling, to train and test the CDSS using 130 scenarios of simulated nursing home residents generated by Generative Pre-Trained Transformer 4.0. Usability was assessed using the System Usability Scale (Brooke, 1996) and a questionnaire by Lund (2001) evaluating usefulness, satisfaction, and ease of use. The System Usability Scale (SUS) yielded a mean score of 60 (SD = 3.65), while the USE score was 6.62 (SD = 0.55), with high ratings for ‘ease of learning.’ Usability issues were also identified through heuristic evaluation with nursing faculty experts. All ratings fell within a range of 5 to 7, indicating overall positive feedback. The CDSS has the potential to significantly enhance care quality, marking a pivotal advancement in applying healthcare technology to support nursing professionals. Future research should reflect on a more nursing care-specific application in developing and applying new technology in long term care settings

